# Influence of residual force enhancement and elongation of attached cross‐bridges on stretch‐shortening cycle in skinned muscle fibers

**DOI:** 10.14814/phy2.13477

**Published:** 2017-11-28

**Authors:** Atsuki Fukutani, Venus Joumaa, Walter Herzog

**Affiliations:** ^1^ Human Performance Laboratory Faculty of Kinesiology University of Calgary Calgary Alberta Canada; ^2^ Japan Society for the Promotion of Science Chiyoda‐ku Tokyo Japan; ^3^ Research Organization of Science and Technology Ritsumeikan University Kusatsu Shiga Japan

**Keywords:** Elastic energy, muscle contraction, muscle physiology, titin

## Abstract

Increased muscle force during stretch‐shortening cycles (SSCs) has been widely examined. However, the mechanisms causing increased muscle force in SSCs remain unknown. The purpose of this study was to determine the influence of residual force enhancement and elongation of attached cross‐bridges on the work enhancement in SSCs. For the Control condition, skinned rabbit soleus fibers were elongated passively from an average sarcomere length of 2.4 to 3.0 *μ*m, activated and then actively shortened to 2.4 *μ*m. For the Transition condition, fibers were elongated actively from an average sarcomere length of 2.4 to 3.0 *μ*m. Two seconds after the end of active lengthening, fibers were actively shortened to 2.4 *μ*m. In the SSC condition, fibers were lengthened actively from an average sarcomere length of 2.4 to 3.0 *μ*m, and then immediately shortened actively to 2.4 *μ*m. Increased muscle force in the SSCs was quantified by the increase in mechanical work during active shortening compared to the mechanical work measured during the purely active shortening contractions. Work enhancement was significantly greater in the SSC compared to the Transition conditions. This difference was associated with the pause given between the active lengthening and shortening phase in the Transition test, which likely resulted in a reduction of the average elongation of the attached cross‐bridges caused by active stretching. Since some work enhancement was still observed in the Transition condition, another factor, for example the stretch‐induced residual force enhancement, must also have contributed to the work enhancement in SSCs.

## Introduction

For a given level of activation, a muscle's force depends on its instantaneous length (Edman 1966; Gordon et al. 1966), shortening/lengthening velocity (Gasser and Hill 1924; Hill 1938), and contractile history (Abbott and Aubert 1952). Importantly, the steady‐state isometric force after active shortening is smaller while the isometric force after active lengthening is greater than that attained in a purely isometric contraction at the corresponding length and activation (Abbott and Aubert 1952). This history dependence of active force production is referred to as (residual) force depression, and (residual) force enhancement, respectively (Maréchal and Plaghki 1979; Edman et al. 1993; Herzog and Leonard 1997; Herzog et al. 2006). The force enhancement property might be expected to contribute to the work enhancement observed in stretch‐shortening cycles (SSCs) (Cavagna et al. 1968; Bosco et al. 1982).

Currently, work enhancement in SSCs is thought to be caused by a stretch‐induced reflex response (Nichols and Houk 1973; Dietz et al. 1979), tendon elongation (Finni et al. 2001; Kawakami et al. 2002), pre‐activation of muscles (Bobbert et al. 1996; Bobbert and Casius 2005), and residual force enhancement (Edman et al. 1978; Joumaa et al. 2008a). Recently, Fukutani et al. (2015, 2016, 2017) found that muscle forces in the active shortening phase were greater in experiments involving SSCs than pure shortening contractions and SSCs without pre‐activation. They speculated that residual force enhancement, and an increase in the extension of attached cross‐bridges (an increase in Huxley's “x”‐distance, Huxley 1957) in the stretch phase of the SSCs contributed to the increased work observed in their study. However, since they tested human plantar‐flexor and knee‐extensor muscles (Fukutani et al. 2015, 2016, 2017), the influence of storage and release of elastic energy in the tendon on the work enhancement in the SSC, could not be excluded (Finni et al. 2001; Kawakami et al. 2002).

The purpose of this study was to determine the effect of residual force enhancement and elongation of attached cross‐bridges on the work enhancement in SSCs. We used skinned fibers to exclude tendon and activation effects as confounding factors. Therefore, we could isolate the effects of residual force enhancement and cross‐bridge extension as the two primary factors contributing to work enhancement in SSCs. Activated fibers were stretched and then shortened with and without a transition phase. Since cross‐bridge cycling is fast, elongated cross‐bridges will quickly detach when a transition phase is provided, thus minimizing their effect on work enhancement.

## Methods

### Sample preparation

New Zealand white rabbits were euthanized according to a protocol approved by the University of Calgary's Life Sciences Animal Ethics Committee. Strips of soleus muscle were harvested and tied to wooden sticks to preserve in situ sarcomere length. Muscle strips were then placed in a rigor‐glycerol solution with protease inhibitors (Complete, Roche Diagnostics, Montreal, Quebec, Canada) to alter the permeability of the muscle fibers and the structure of the connective tissue. Strips were stored at −20° for at least 21 days. On the day of the experiments, a single fiber of the soleus was isolated using fine forceps and a dissecting microscope (SMZ1500; Nikon, Tokyo, Japan). The isolated fiber was transferred to an experimental chamber (Models 802B; Aurora Scientific, Ontario, Canada) containing a relaxing solution with protease inhibitors. One end of the fiber was attached to a force transducer (Model 400A; Aurora Scientific, Ontario, Canada), the other end to a length controller (Model 308B; Aurora Scientific, Ontario, Canada). Sarcomere length was measured using a He‐Ne laser‐based diffraction system (1508P‐1; JDSU, California, the United States). Fiber length was measured using a microscope (Stemi 2000; Zeiss, Oberkochen, Germany). All experiments were conducted at room temperature (19–23°C). This experimental set up was similar to that used previously (Joumaa and Herzog 2013, 2014).

### Mechanical tests

**Figure 1:**
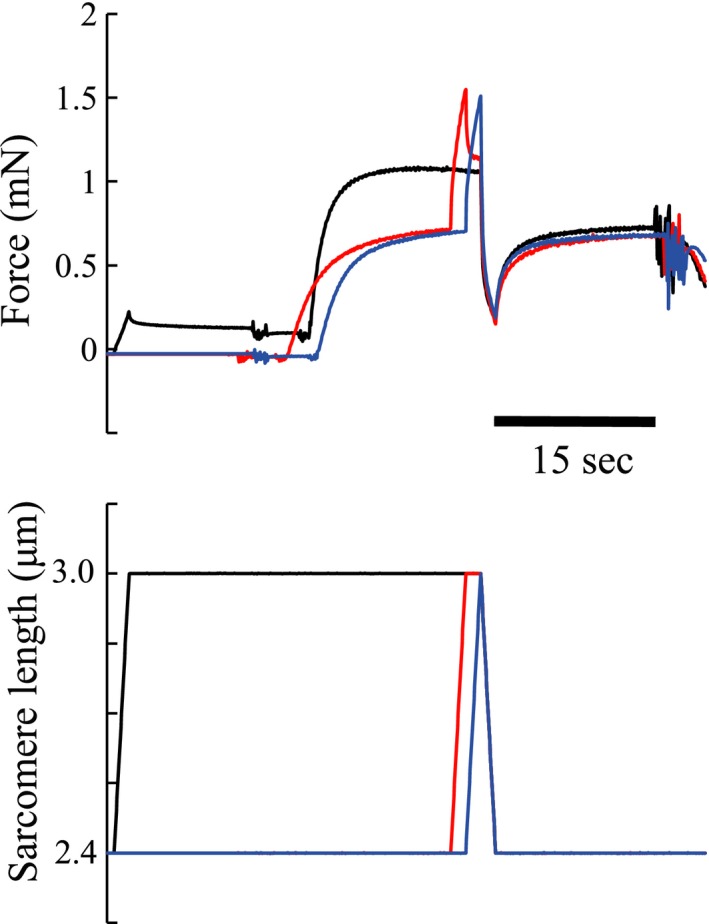
Typical force and sarcomere length changes as a function of time for the three experimental conditions of Experiment 1. The black line indicates the control condition. The red line indicates the transition condition. The blue line indicates the stretch‐shortening cycle (SSC) condition.

We performed two experiments on a total number of 49 fibers. Experiment 1 (*n* = 49 fibers) consisted of three trials. In trial 1 (Control condition, see Figs. 1, 3, black line), fibers were stretched passively from an average sarcomere length of 2.4 *μ*m to an average sarcomere length of 3.0 *μ*m in 2 s. Fibers were then activated, and once maximal isometric force was reached, fibers were shortened to an average sarcomere length of 2.4 *μ*m in 2 s. Fibers were deactivated 15 s after the end of the shortening phase. In trial 2 (Transition condition, see Figs. 1, 3, red line), fibers were activated at an average sarcomere length of 2.4 *μ*m, stretched to 3.0 *μ*m in 2 s, held isometric for 2 s, shortened back to an average sarcomere length of 2.4 *μ*m in 2 s, and held isometrically for another 15 s. Trial 3 (SSC condition, see Figs. 1, 3, blue line) was identical to trial 2, except that there was no pause between the stretch and the shortening phase. Trial 1 was always conducted first, while trials 2 and 3 were conducted in random order. A minimum rest of 3 min was given between trials. Trials 1–3 were also performed purely passively, without fiber activation. The active force in trials 1–3 was then calculated by subtracting the force in the passive trials from the total force of trials 1–3. All data were collected at a rate of 10,000 Hz.

**Figure 2:**
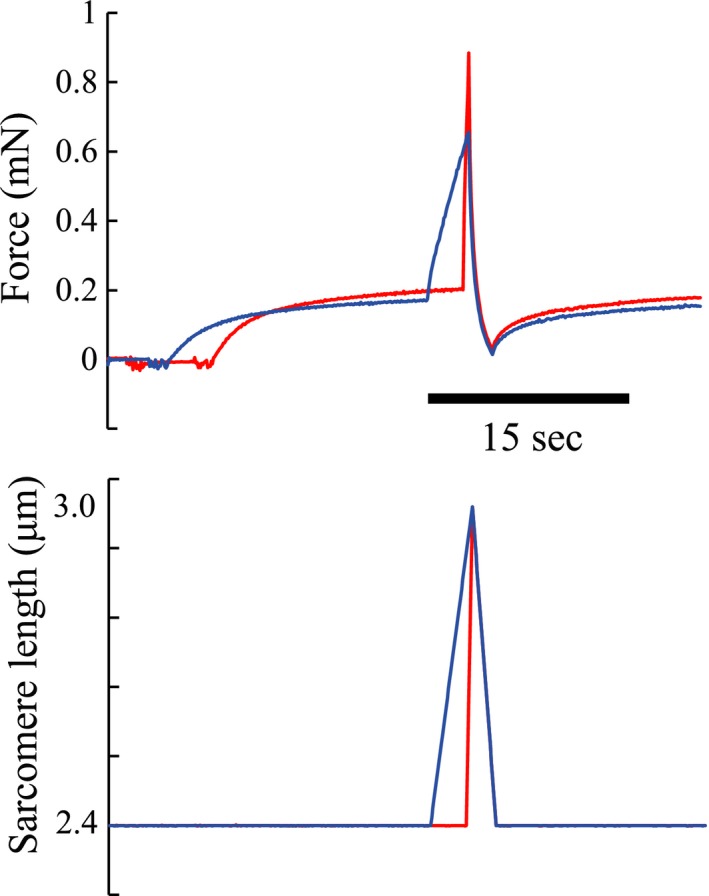
Typical time courses of force and sarcomere length for the two conditions of Experiment 2. The red line indicates the fast condition. The blue line indicates the slow condition.

Experiment 2 (*n* = 45 fibers) was identical to experiment 1, except that the stretch velocities in the SSC cycles were fast (stretch phase completed in 0.5 s instead of 2 s as done in Experiment 1), or slow (stretch phase completed in 3.5 s) (Fig. 2). The sequence of these two tests was randomized and a minimum of 3 min rest was given between tests.

### Analyses and measurements

The primary outcome measure for experiment 1 and 2 was the work performed in the shortening phase. The work during muscle fiber shortening was calculated by integrating fiber force over the fiber shortening distance. In addition, force before the active stretch, at the end of the active stretch, at the onset of active shortening, at the end of active shortening, and 15 s after the active shortening, were measured.

### Statistics

In Experiment 1, differences in the mechanical work were evaluated using a one‐way repeated measures analysis of variance (ANOVA) with the primary factor “condition” (Control, Transition, and SSC conditions). Differences in force were determined using a two‐way repeated measures ANOVA with the primary factors “condition” (Control, Transition, and SSC conditions) and “time” (onset of active shortening, end of active shortening, and 15 s after the end of active shortening). Forces prior to and after the active stretch were compared between the Transition and SSC conditions using a paired *t*‐test.

For Experiment 2, a paired *t*‐test was conducted to determine possible differences in mechanical work between the Fast and Slow stretch conditions. Forces at different time points were evaluated using a two‐way repeated measures ANOVA with the primary factors “condition” (Fast and Slow conditions) and “time” (before active stretch, end of active stretch, onset of active shortening, end of active shortening, and 15 s after the end of active shortening).

The effect size for the ANOVAs was determined as the partial *η* ^2^, and effect size for the post hoc tests was calculated as Cohen's *d*. Statistical analyses were performed using SPSS (version 20; IBM, Tokyo, Japan), with the level of significance set at *P* < 0.05.

### Solutions

The relaxing solution contained (in mmol/L) 170 potassium propionate, 2.5 magnesium acetate, 20 MOPS, 5 K_2_EGTA, and 2.5 ATP, pH 7.0. The washing solution contained (in mmol/L) 185 potassium propionate, 2.5 magnesium acetate, 20 MOPS, and 2.5 ATP, pH 7.0. The activating solution contained (in mmol/L) 170 potassium propionate, 2.5 magnesium acetate, 10 MOPS, 2.5 ATP and free Ca^2+^ buffered with EGTA (CaEGTA and K_2_EGTA mixed in order to obtain a pCa value of 4.2), pH 7.0. One tablet of protease inhibitors was added to each 100 mL of relaxing solution.

## Results

### Experiment 1

**Figure 3:**
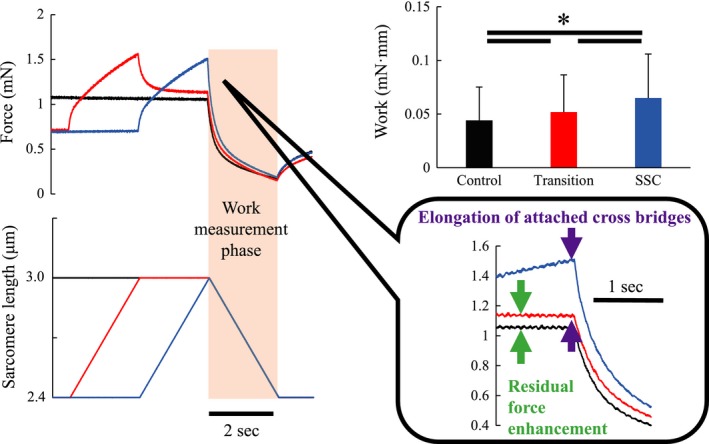
Mechanical work done during the concentric contraction phase in Experiment 1. *Indicates a significant difference between conditions (*P* < 0.05). The black line indicates the control condition. The red line indicates the Transition condition. The solid line indicates the stretch‐shortening cycle (SSC) condition.

Mechanical work was greater in the SSC condition than in the Transition condition (*P* < 0.001, Cohen's *d* = 0.35) and was greater in the Transition condition than the Control condition (*P* < 0.001, Cohen's *d* = 0.23, Fig. 3).

**Figure 4:**
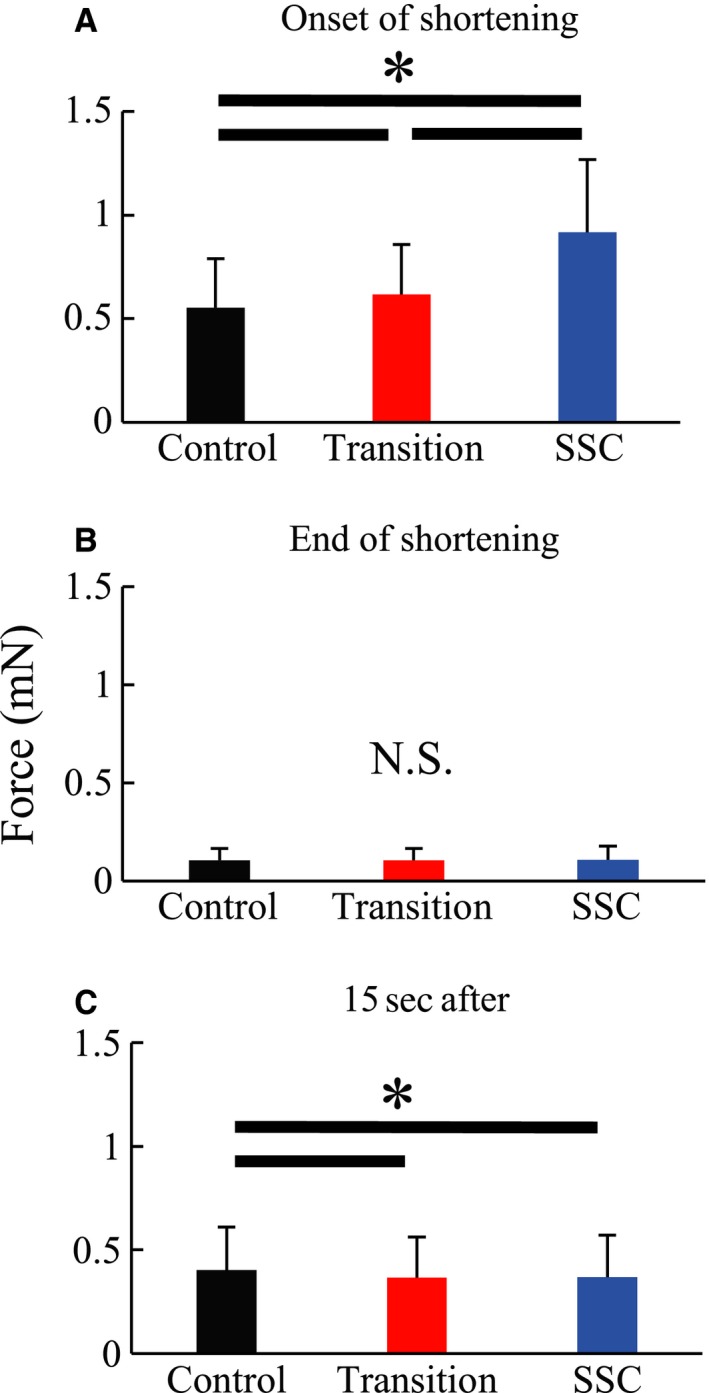
Force at the onset of active shortening (A), at the end of active shortening (B), and 15 s after the active shortening (C) in Experiment 1. *Indicates a significant difference between conditions (*P* < 0.05).

Force at the onset of shortening was significantly greater in the SSC than the Transition condition (*P* < 0.001, Cohen's *d* = 1.00), and was significantly greater in the Transition than the Control condition (*P* < 0.001, Cohen's *d* = 0.27) (Fig. 4A). Force at the end of shortening was the same for all conditions (*P* = 0.745, Cohen's *d* = 0.05 for Control vs. Transition, *P* = 0.491, Cohen's *d *= 0.07 for Control vs. SSC, *P* = 0.152, Cohen's *d* = 0.11 for Transition vs. SSC) (Fig. 4B). Force at 15 s following shortening was significantly greater in the Control than the Transition and SSC conditions (*P* < 0.001, Cohen's *d* = 0.20 for Control vs. Transition, *P* < 0.001, Cohen's *d* = 0.16 for Control vs. SSC), while there was no difference in force at this time point between the Transition and SSC conditions (*P* = 0.774, Cohen's *d* = 0.03) (Fig. 4C).

**Figure 5:**
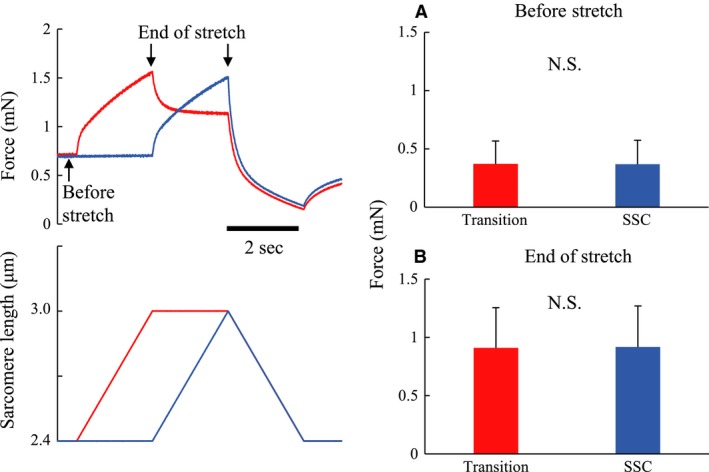
Force before the active stretch (A) and after the active stretch (B) in Experiment 1. The red line indicates the Transition condition. The blue line indicates the stretch‐shortening cycle (SSC) condition.

There was no difference in force prior to and at the end of the stretch phase between the Transition and SSC conditions (*P* = 0.768, Cohen's *d* = 0.02 prior to stretch, and *P* = 0.226, Cohen's *d* = 0.03 at the end of stretch) (Fig. 5).

**Figure 6:**
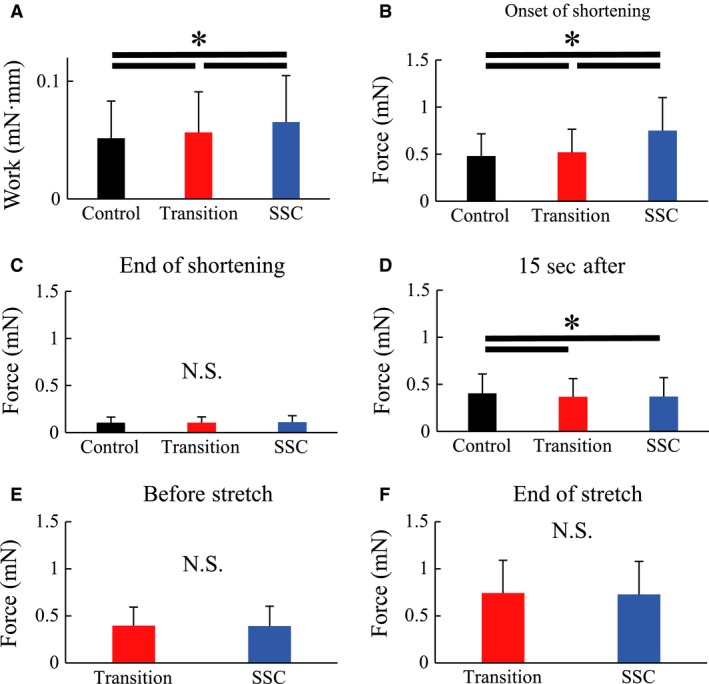
Active work and active force (passive force was subtracted from total force) in Experiment 1. (A) Mechanical work done during the concentric contraction phase. Force at the onset of active shortening (B) and at the end of active shortening (C). (D) Force 15 s after the end of active shortening. Force before active elongation (E) and at the end of active elongation (F). *Significant difference between conditions (*P* < 0.05). Note that the statistical results were identical with those in Figures 3, 4, 5 (i.e., the results of total force).

The results for the active forces (Fig. 6) were the same as those obtained for the total forces (Fig. 3, 4, 5).

### Experiment 2

**Figure 7:**
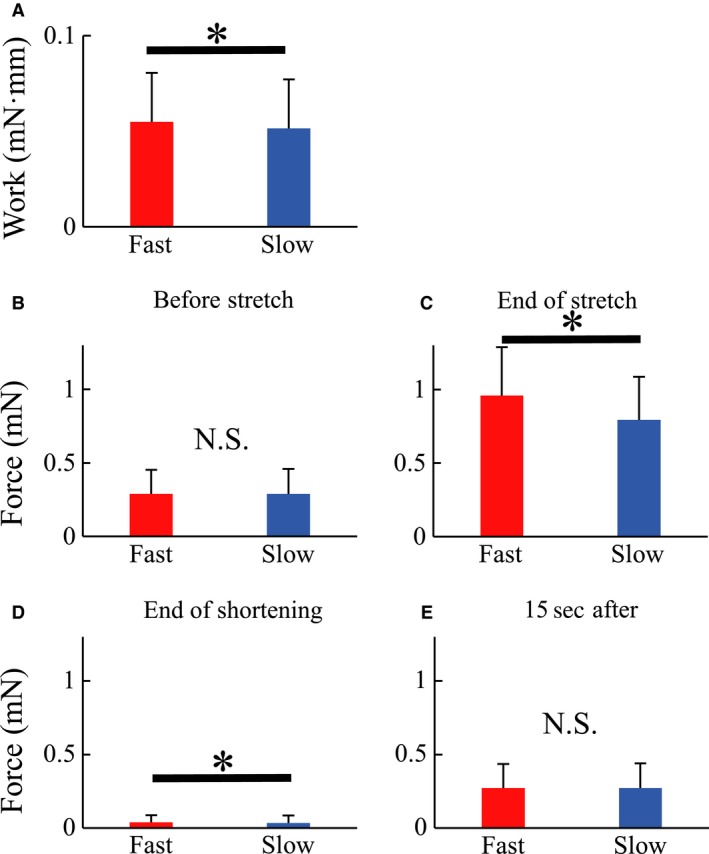
Work and force in Experiment 2. (A) Mechanical work done during the concentric contraction phase. Force before active elongation (B) and at the end of active elongation (C). (D) Force at the end of the active shortening. (E) Force 15 s after the end of active shortening. *Significant difference between conditions (*P* < 0.05).

Work performed during the shortening phase of the SSC was significantly greater in the Fast than the Slow condition (*P* < 0.001, Cohen's *d* = 0.14) (Fig. 7A).

Force at the end of the stretch phase in SSC (and thus also the onset of shortening) was significantly greater in the Fast than the Slow condition (*P* < 0.001, Cohen's *d* = 0.53) (Fig. 7C). Although force at the end of shortening was significantly greater in the Fast than the Slow condition (*P* = 0.005, Cohen's *d* = 0.06) (Fig. 7D), the absolute difference (0.003 mN) was small and corresponded to 1% of the isometric force measured prior to the stretch phase (0.289 mN) (Fig. 7B). Force at 15 s following the end of the shortening phase was the same for all conditions (*P* = 0.926, Cohen's *d* = 0.02) (Fig. 7E). Force prior to stretching was the same for all conditions (*P* = 0.827, Cohen's *d* < 0.01).

## Discussion

The purpose of this study was to investigate work enhancement in skinned muscle fibers and determine the contribution of residual force enhancement and cross‐bridge elongation on work enhancement in SSCs. We achieved this purpose using skinned fibers and adding a transition phase to the SSCs. According to the cross‐bridge theory, one would expect the average cross‐bridge extensions to be greater following fiber stretching compared to the purely isometric conditions. However, by adding a pause of sufficient time, one would expect the average cross‐bridge extensions to become that of the isometric control conditions. Accordingly, we observed more work in the SSC than the Transition conditions (Fig. 3). This work difference may be explained by the loss of elastic energy stored in the extended cross‐bridges for the Transition condition. Nevertheless, the work in the Transition conditions was greater than the Control conditions (Fig. 3), indicating that cross‐bridge extension was not the only factor contributing to the enhanced work in SSCs, but that the residual force enhancement may have contributed too.

When activated muscle fibers are stretched, the attached cross‐bridges are stretched as well, and so produce greater force (Huxley 1957). This change in cross‐bridge force may contribute to the SSC effect. This observation is substantiated by the greater forces at the onset of shortening in the SSC compared to the Transition condition (Fig. 4A). However, the elongated cross‐bridges cannot provide additional force/work for great periods of time or shortening distances as attached cross‐bridges detach quickly from actin filaments (Huxley and Simmons 1971), and their stored elastic energy is lost (Bosco et al. 1982; Wilson et al. 1991).

Work enhancement in the Transition condition was smaller than in the SSC condition, which may be explained by the loss of elastic energy stored in cross‐bridges attached at the end of active stretching. However, work in the Transition condition was greater than that obtained in the Control condition (Fig. 3), indicating that factors other than cross‐bridge extension must have contributed to the enhanced work. A possible factor contributing to this increased work is the residual force enhancement (Abbott and Aubert 1952; Joumaa et al. 2008a). Residual force enhancement has been shown to be long lasting and is largely explained by the engagement of a passive structural element (Noble 1992; Forcinito et al. 1998), likely titin (Joumaa et al. 2008b; Leonard and Herzog 2010). The energy stored in this passive structural element is not lost, but would be expected to contribute to the enhancement of force and work throughout the entire range of shortening (Seiberl et al. 2015; Fortuna et al. 2017). If titin behaves viscoelastically, elastic energy is dissipated to some extent during the transient period. However, because titin is thought to behave essentially elastically within the physiological range of muscle excursion (Kellermayer et al. 1997), we would not expect energy to be lost from titin in the transient period of SSCs.

If elastic energy stored in attached cross‐bridges contributes to the SSC effect, the SSC effect should become larger when the speed of active lengthening becomes higher, because the magnitude of the elastic energy stored in the attached cross‐bridges is larger. As expected, and shown previously (Edman et al. 1978; Sugi and Tsuchiya 1988;. Lombardi and Piazzesi 1990), force at the end of the active stretch was higher (Fig. 7C), and resulted in an increase in the mechanical work for the Fast compared to the Slow condition (Fig. 7A). The influence of the residual force enhancement would be expected to be similar for the Fast and Slow conditions because the magnitude of the residual force enhancement is essentially unaffected by the speed of muscle stretching (Edman et al. 1982; Sugi and Tsuchiya 1988; Lee and Herzog 2002). Thus, it is reasonable to assume that the observed larger mechanical work in the Fast compared to the Slow condition (Cavagna et al. 1968; Bosco et al. 1981) was caused by the increased elastic cross‐bridge energy.

For submaximal contractions, the proportion of attached cross‐bridges is smaller than for maximally activated muscles. Thus, the effect of elongation of attached cross‐bridges would be expected to be smaller as well in submaximal compared to maximal contractions. However, the amount of the absolute residual force enhancement has been shown to remain similar for maximal and submaximal contractions (De Ruiter et al. 2000; Minozzo and Rassier 2013), because residual force enhancement is largely independent of cross‐bridge kinetics and is primarily explained by the engagement of passive structural elements (Leonard and Herzog 2010; Powers et al. 2014). Therefore, the work enhancement observed in this study for maximal contractions might be similar for submaximal contractions, and thus, the relative effect of work enhancement in SSCs might be more pronounced, and potentially play a greater functional significance at submaximal compared to maximal muscle contractions.

## Conclusions

Based on the results of this study, we conclude that residual force enhancement and the elastic energy stored in cross‐bridges following active muscle stretching are contributors to the enhanced work observed following SSCs.

## Conflict of Interest

The authors report no competing interests for this work.
